# Powdery Mildew-Induced Hormonal and Photosynthetic Changes in Barley Near Isogenic Lines Carrying Various Resistant Genes

**DOI:** 10.3390/ijms21124536

**Published:** 2020-06-25

**Authors:** Diana Saja, Anna Janeczko, Balázs Barna, Andrzej Skoczowski, Michał Dziurka, Andrzej Kornaś, Gábor Gullner

**Affiliations:** 1 Polish Academy of Sciences, The Franciszek Górski Institute of Plant Physiology, Niezapominajek 21, 30-239 Krakow, Poland; d.saja@ifr-pan.edu.pl (D.S.); andrzej.skoczowski@up.krakow.pl (A.S.); m.dziurka@ifr-pan.edu.pl (M.D.); 2 Plant Protection Institute, Centre for Agricultural Research, Herman Ottó út 15, 1022 Budapest, Hungary; barna.balazs@agrar.mta.hu (B.B.); gullner.gabor@agrar.mta.hu (G.G.); 3 Institute of Biology, Pedagogical University of Krakow, Podchorążych 2, 31-054 Krakow, Poland; andrzej.kornas@up.krakow.pl

**Keywords:** barley, *Blumeria graminis*, chlorophyll *a* fluorescence, leaf reflectance, phytohormones, resistance genes

## Abstract

The present work focused on the characterization of some physiological mechanisms activated upon powdery mildew inoculation of the susceptible barley cultivar Ingrid and its near-isogenic lines (NILs) carrying various resistant genes (*Mla*, *Mlg* and *mlo*). After inoculation with *Blumeria graminis* f. sp. *hordei* (*Bgh*), measurements of leaf reflectance and chlorophyll *a* fluorescence were performed 3 and 7 day post-inoculation (dpi), while hormone assays were made 7 dpi. *Bgh*-inoculated resistant genotypes were characterized by lowered leaf reflectance parameters that correlated with carotenoids (CRI) and water content (WBI) in comparison to inoculated Ingrid. The PSII activity (i.e., Fv/Fm, ETo/CSm and P.I._ABS_) strongly decreased in susceptible Ingrid leaves when the disease symptoms became visible 7 dpi. In Mla plants with visible hypersensitive spots the PSII activity decreased to a lesser extent. Inoculation resulted in a very slight decrease of photosynthesis at later stage of infection in Mlg plants, whereas in resistant mlo plants the PSII activity did not change. Chlorophyll *a* fluorescence measurements allowed presymptomatic detection of infection in Ingrid and Mla. Changes in the homeostasis of 22 phytohormones (cytokinins, auxins, gibberellins and the stress hormones JA, SA and ABA) in powdery mildew inoculated barley are discussed in relation to resistance against this biotrophic pathogen.

## 1. Introduction

Barley is one of the most important cereals grown in the world. In Poland, its cultivation area covers about 12% of the total area dedicated to all cereals. Barley is used as food for humans and a livestock feed for animals, as well as for preparation of alcoholic and non-alcoholic beverages. Apart from unfavorable meteorological and soil conditions, large losses in barley yield are caused by fungal diseases. Powdery mildew caused by *Blumeria graminis* f. sp. *hordei* (*Bgh*) is one of the most devastating barley diseases in the world [1]. This biotrophic fungus attacks the leaves and covers them with white mycelia. Yield losses due to this fungus are usually around 10–20%, but may exceed even 50%. This is due to loss of functional green leaf surface and to reduction in grain weight and the number of grains in the tiller, which is important in the production of malt [2]. The disease is particularly prevalent in temperate zones [3]. The occurrence of new races of (*Bgh*) poses a critical problem, since it can destroy the resistance of barley. This fungus has a large number of traits that support rapid evolution, such as a large number of asexual haploid spores, the ability to genetic recombination during the growing season and long-range propagation in the air [4]. Fungicide treatments are used to support the control this disease, however, application of fungicides has been increasingly limited by development of fungicide resistance in pathogens and by their potential harmful effects on human health and the environment [2]. The use of genetically resistant barley varieties is one of the most economically and environmentally desirable strategies for control, and therefore research is particularly important in this area. It is essential that new cultivars be bred with broad-spectrum resistance genes. Effective models for studies of mechanisms of barley resistance to powdery mildew are near-isogenic lines with various types of resistance genes, like *Mla*, *Mlg* and *mlo*. The Mla type of resistance is race specific, with visible hypersensitive spots, the Mlg resistance is also race specific and hypersensitive type but can be characterized with invisible single cell death. On the other hand, mlo type of resistance is race non-specific, effective against all barley powdery mildew races, and not hypersensitive, but based on papilla formation [5]. The main novelty in the use of these different barley resistance genes is that they confer different type of resistance to powdery mildew. The strongest, which stops the development of the pathogen at very early stage, is *mlo* gene, while *Mla* allows the most fungal development and *Mlg* is in between. These differences are well visualized on scheme by Hückelhoven et al. [6]. *Mla* and *mlo* are two of the most effective and widely deployed powdery mildew resistance genes used in barley breeding [7]. The mlo resistance is monogenic [8]. The *mlo* is a broad-spectrum resistance gene on chromosome 4H. It was identified in Ethiopian landraces but was later reconfirmed in multiple forms from mutation studies [8]. Plants expressing *mlo*-alleles were first released on the market in 1979, but the resistance of these varieties still remains highly effective. The *Mla* is one of the most precisely characterized gene that confers resistance to powdery mildew. The gene has 32 known alleles and represents a true allelic series on chromosome 1H [9].

Earlier physiological studies revealed an elevated ethylene production and an increased membrane permeability in leaves of the susceptible barley cultivar Ingrid at 3 days post inoculation (3dpi) with powdery mildew [10]. Probably due to the limited number of affected cells, no changes of ethylene production were detected in resistant, near-isogenic Ingrid mutants carrying the *Mlg* and *mlo* genes and only a slight increase was found in Mla leaves even at 4 dpi. Activities of antioxidant enzymes such as peroxidase, superoxide dismutase, glutathione S-transferase, ascorbate peroxidase and glutathione reductase were markedly induced by fungal infection in susceptible Ingrid leaves at 5–7 dpi. Similar, but less intense pathogen-induced changes were detected in inoculated leaves of Mla line that showed hypersensitive cell death upon inoculation, and to an even lesser extent, in the Mlg and mlo lines, where no visible symptoms accompanied the incompatible interaction. Finally, Harrach et al. [10] confirmed the hypothesis that hydrogen peroxide may play a pivotal role in the resistance of barley to powdery mildew since in contrast to resistant barley lines, H_2_O_2_ level decreased in susceptible Ingrid leaves after inoculation. Further studies proved the exposition of the above near-isogenic barley lines to heat shock induced susceptibility against powdery mildew [11]. The elevated susceptibility by heat treatment was confirmed also by microscopic observations in susceptible cv. Ingrid as well as in its near-isogenic Mla, Mlg even mlo lines.

Fungal infections can cause various changes in crucial plant processes such as photosynthesis or respiration and thus have a general impact on whole plant metabolism. As for light reactions of photosynthesis, pathogen infections affect the efficiency of photosystems (PS) that is mainly connected to the ultrastructure and functionality of chloroplasts [12,13]. In addition the oxygen- evolving complex of the photosynthetic electron transport chain was identified as a target of pathogens (viruses) [14,15,16]. As for other metabolic alterations (in plants affected by pathogens), changes in hormonal homeostasis are one of important line of defense. Therefore the aim of our present studies was to describe how powdery mildew inoculation of wild type cv. Ingrid and its three near-isogenic lines (Mla, Mlg and mlo) influences leaf physiological properties including photosynthetic efficiency as well as leaf homeostasis of a wide range of hormones including their precursors and conjugates.

## 2. Results and Discussion

### 2.1. Appearance of Disease Symptoms

**Figure 1:**
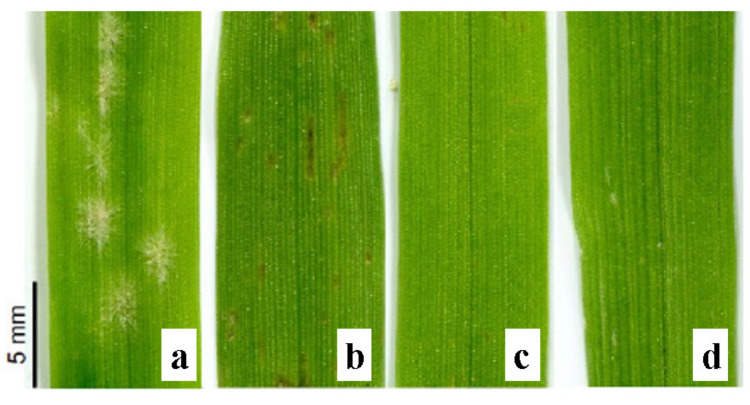
Barley leaves after powdery mildew inoculation at 7 days post inoculation (dpi): cultivar Ingrid with visible symptoms (**a**), as well as its near isogenic lines-Mla with visible symptoms (**b**), Mlg (**c**) and mlo (**d**).

No visible disease symptoms were observed on the leaves of any tested genotypes at 2 or 3 dpi. The first, very mild symptoms appeared at 4 dpi on leaves of Ingrid and Mla lines, but not of other lines. Full symptom development was observed at 6–7 dpi in the case of Ingrid (susceptible cultivar) and Mla line (necrotic spots). No symptoms were observed on leaves of Mlg and mlo plants (Figure 1).

### 2.2. Measurements of Leaf Reflectance

**Figure 2:**
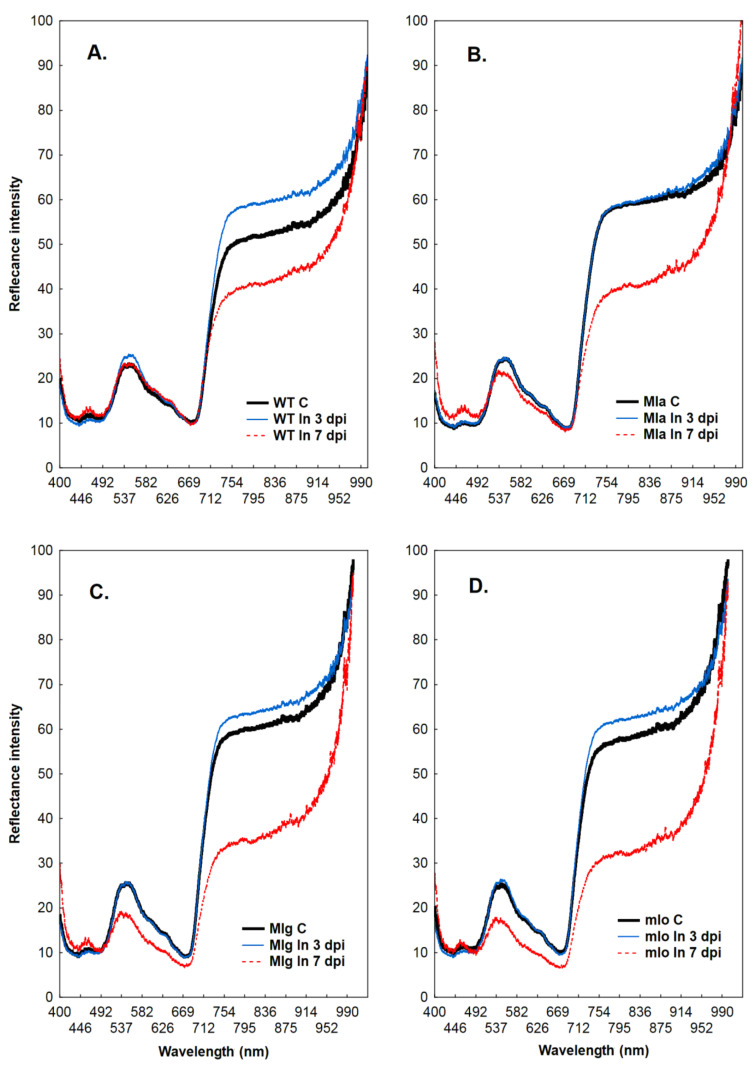
Reflectance intensity of barley leaves of the wild type cultivar Ingrid (panel **A**) and its near-isogenic lines Mla (**B**), Mlg (**C**), and mlo (**D**). Abbreviations: WT, wild type Ingrid; C, control leaves; In, leaves inoculated with powdery mildew: 3 dpi or 7 dpi (leaves 3 and 7 days post inoculation with powdery mildew). Each curve represents the average of measurements made on 10 plants. Since the reflectance values of not inoculated control leaves were similar at time points corresponding to 3 and 7 dpi they were summed up and shown as one control.

The reflectance intensity of the wild type cultivar Ingrid in the range of 400–700 nm was similar between powdery mildew inoculated and control plants at both 3 and 7 dpi (Figure 2A). In the near-isogenic Mla, Mlg and mlo lines the reflectance was comparable in this range between control and inoculated at 3 dpi, while decreased reflectance intensity was observed in the infected leaves at 7 dpi (Figure 2B–D). In the range of 700–1000 nm an increased reflectance intensity was revealed in the inoculated leaves of Ingrid, Mlg and mlo plants (but not in Mla) at 3 dpi (Figure 2A,C,D, blue line). However, the reflectance intensity strongly decreased in all inoculated lines at 7 dpi in comparison to respective controls, but the smallest decrease was observed in wild type Ingrid leaves (Figure 2A‒D, red line).

The spectral quality of light reflected from leaves depends on their pigment composition (range 400–700 nm), but also on physical properties of leaf surface (infrared range >700 nm). A lot of studies have been done to link changes in reflection spectrum with plant responses to physiological stress [17,18,19,20]. A wide range of abiotic and biotic stress factors were studied in different plant species ranging from grasses to deciduous and coniferous trees. In all cases, differences in reflectance intensities between control and stressed plants within the range 400–700 nm wavelength were observed as increased reflectance at wavelengths near 700 nm.

In summary, highly consistent changes in leaf reflectance, which occur in plants under stress especially in green-yellow and far-red spectra, can be explained by the stress-induced decrease in chlorophyll concentration in the leaves [20]. Generally, it is expected that stress causes a loss in chlorophyll content. However, the strongly decreased reflection intensities in the 500–700 nm range in Mlg and mlo genotypes at 7 dpi indicates a significant increase in the amount of chlorophyll in leaves of these lines as compared to control and infected leaves at 3 dpi (Figure 2C,D; red line).

**Figure 3:**
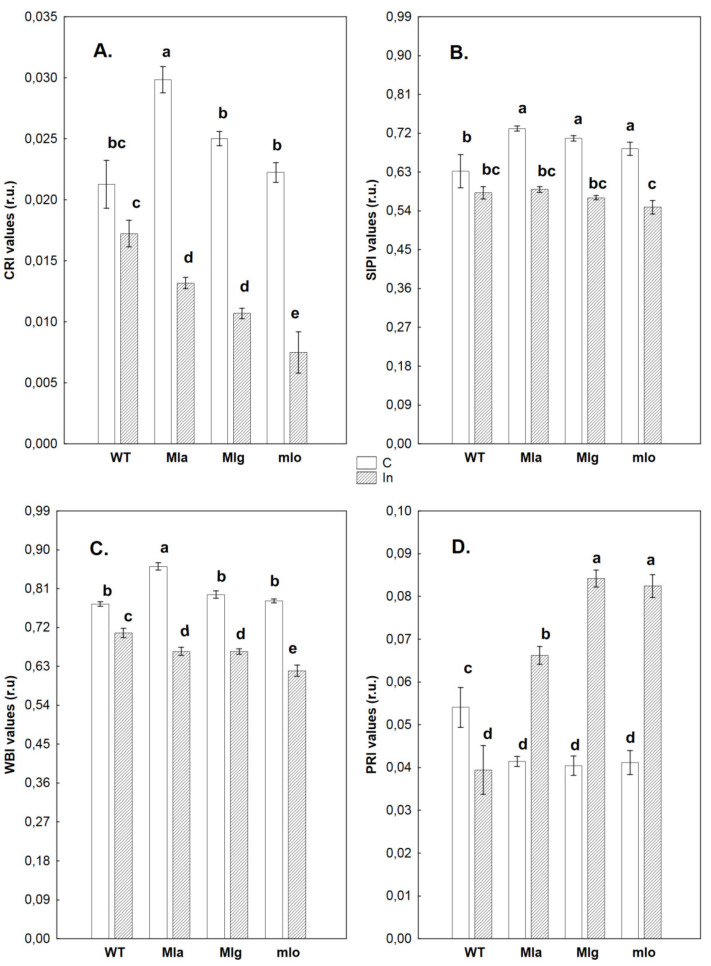
Leaf reflectance parameters showing changes in the pigment composition and water content in the leaves of the wild type barley cultivar Ingrid and its near isogenic lines Mla, Mlg, mlo. Four different parameters are shown, (**A**): carotenoid content (CRI); (**B**): ratio of carotenoids to chlorophyll *a* content (SIPI); (**C**): water content (WBI); (**D**): photochemical reflectance index (PRI). Abbreviations: WT, wild type Ingrid; C, control leaves; In, powdery mildew inoculated leaves at 7 days post inoculation (7 dpi). Mean values from 10 repetition ± SD. Values marked with the same letters do not differ significantly at *p* ≤ 0.05 according to the Duncan’s test.

The curves shown on Figure 2 allowed us to calculate leaf reflectance parameters characterizing the amount of carotenoids (CRI), the ratio of carotenoids to chlorophyll *a* content (SIPI), the leaf water content (WBI) and the photochemical reflectance index (PRI) (Figure 3). The changes in these parameters were not significant in any inoculated leaves at 3 dpi (data not shown). The highest carotenoid content (CRI value) was observed in leaves of not inoculated Mla plants but in rest of lines, including Ingrid, this parameter reached lower values (Figure 3A). After powdery mildew inoculation of Mla, Mlg and mlo lines, their carotenoid contents, expressed indirectly as CRI, were lower in comparison to their respective not inoculated controls. Interestingly, the CRI value of inoculated Ingrid did not change statistically significantly as compared with control (only a tendency of decrease was observed). In inoculated Mla, Mlg and mlo plants, the CRI values were finally significantly lower than that of inoculated Ingrid (the lowest CRI was in inoculated mlo) (Figure 3A).

According to Wang et al. [21], powdery mildew infection of rubber tree was connected to an increase of carotenoids content. Therefore, it is possible that the defense reaction of powdery mildew resistant plants would be an opposite reaction‒a reduction of carotenoid content following inoculation. This phenomenon can be seen in Mla, Mlg, and especially, in the strongest form, in the mlo line. The sustained high carotenoid level in infected Ingrid at a level of not inoculated plants might be the result of weak defense mechanisms or even caused by the fungal production of carotenoids. An earlier report of Harrach et al. [10], did not exclude the possibility that the powdery mildew fungus itself produces antioxidant substances or enzymes to defend themselves against reactive oxygen species generated during the infection process. On the other hand, it is known that fungi (i.e., *Fusarium fujikuroi)* can produce carotenoids [22] among other as defense against oxidative stress [23]. In the light of this interpretation, the low content of carotenoids as substances with an antioxidant capacity in inoculated mlo plants could contribute to antifungal resistance by allowing plants to limit pathogen growth by a higher production of reactive oxygen species like H_2_O_2_ due to a low level of antioxidants [10].As for the SIPI parameter (ratio of carotenoids to chlorophyll *a* content, Figure 3B), generally the values in the control group and the pattern of changes (not inoculated vs. inoculated plants) were very similar for all tested plants as in case of CRI. In inoculated Ingrid the SIPI value was practically unchanged in comparison to Ingrid control. In other barley lines, SIPI values were lower after inoculation in comparison to respective controls. Finally, there was no significant difference in SIPI values between all four inoculated lines at 7 dpi.

Water content in control and inoculated plants was expressed by water band index (WBI) values (Figure 3C). Generally, control plants were better hydrated than inoculated ones. Among the control plants, Mla was characterized by the highest water content. Upon inoculation, the water content markedly decreased in all barley lines. Relatively the largest decrease in leaf hydration was observed in the three resistant genotypes. WBI values for green vegetation are usually in the range of 0.8 to 1.2 [24]. In our experiments, WBI values for all barley genotypes was relatively low, just above 0.8 (not inoculated Mla) or below 0.8 (all other inoculated and not inoculated genotypes). Since similarly low WBI values have already been observed in barley [25], low WBI can be a species feature. A decrease in WBI value after inoculation indicates a significant decrease in leaf hydration, which occurred after contact between leaf surface and pathogen. This phenomenon can be explained by different ways. It cannot be excluded that closing stomata as a result of inoculation may limit the suction force of transpiration and, as a consequence, impede the uptake and transport of water. On the other hand, in the case of powdery mildew and rust diseases rather the disruption of epidermis and cuticle is responsible for the elevated transpiration resulting in tissue water loss [26]. Interestingly, inoculated susceptible Ingrid leaves showed the highest water content while highly resistant mlo leaves had the lowest. Decreased WBI was already earlier noted in *Bgh* inoculated barley cultivar Delisa and its 522DK mutant, which were also resistant to powdery mildew [25]. In all tested inoculated genotypes the cuticle was less injured, and water loss was rather inversely proportional to visible symptom appearance. The role of powdery mildew infection in limiting water loss in the susceptible cultivar Ingrid requires further studies.

PRI (photochemical reflectance index) was originally derived as a measure of xanthophyll cycle activity determined using proximal remote sensing of leaves and canopies on a diurnal timescale [27,28]. PRI values may correlate with PS II quantum yield [19]. PRI may also be correlated with epoxidation state and with NPQ, as determined by measuring chlorophyll fluorescence [29,30]. Among not inoculated plants, the highest PRIs were observed in Ingrid while lower values were found in Mla, Mlg and mlo lines, which were comparable to each other (Figure 3D). Interestingly, the opposite situation was found in inoculated plants, where the lowest PRI was observed in Ingrid, and this PRI was even smaller than that of not inoculated Ingrid leaves. However, in inoculated Mla, Mlg and mlo lines the PRI values drastically increased as compared to healthy controls. The PRI value in Mla was a little lower in comparison to both remaining isogenic lines but these plants showed also visible hypersensitive necrotic spots which could have an impact (Figure 1). Interestingly, the PRI results of all tested plants were in some way confirmed by fluorescence measurements and by calculation of the performance index P.I._ABS_ that represents general PSII efficiency (Table S1). The highest P.I._ABS_ reaching 2.26 was noticed in not inoculated Ingrid (see control ‘WT C 7’ in Table S1), while in Mla, Mlg and mlo the corresponding control values ranged between 1.58–1.72. On the other hand, the lowest P.I._ABS_ (0.80) was found in inoculated Ingrid at 7 dpi (similarly as PRI), while this parameter was a little higher (up to 1.10) in hypersensitively reacting Mla (Figure 1). The highest PSII efficiencies, similarly to the highest PRIs, were measured at 7 dpi for uninjured leaf blades of inoculated Mlg and mlo (1.80 and 1.84, respectively, see Table S1). Generally, the PRI values indicate on the effective use of light by the inoculated Mla, Mlg and mlo plants. This may be connected to better protection of photosynthetic light reactions by the xanthophyll cycle and probably allows for more energy to be obtained for defense against the fungus.

The present results fully confirm our previous observations with reflection spectra for wild type barley cultivar Delisa and its brassinosteroid-deficient mutant (527DK) inoculated with powdery mildew [25]. In brief, both genotypes showing no disease symptoms were characterized by an increased foliar chlorophyll content at 7 dpi (a decrease in the reflection intensity in the yellow-green range and at about 700 nm) and a strong decrease in the intensity of reflection in the far-red range as compared to the control. In addition, there was a significant decrease in the amount of carotenoids and a decrease in their hydration after inoculation. At the same time, the PRI markedly increased in inoculated leaves as compared to control ones, which indicates the role of the photosynthetic apparatus in the response/resistance of barley to powdery mildew.

Measurement of leaf reflectance was already used for the study of plant-pathogen interactions, for example to an early detection of viral infection in tomato [31]. This report demonstrated that leaf reflectance measurements allowed presymptomatic detection of *Tomato spotted wilt virus* (TSWV) infection in tomato. In our experiments, the intensity of reflection did not differ between inoculated and healthy barley leaves in the 400–700 nm range, but it was higher in the 700–1000 nm range in the infected leaves at 3 dpi than control (Figure 2). Depending on the genotype, the increase in reflection intensity in the far-red range can be more or less pronounced (Figure 2A–D). Nevertheless, in our opinion, the analysis of reflection changes in the far-red area makes an early, presymptomatic detection of powdery mildew infection possible.

### 2.3. Fast Kinetics of Chlorophyll a Fluorescence

**Figure 4:**
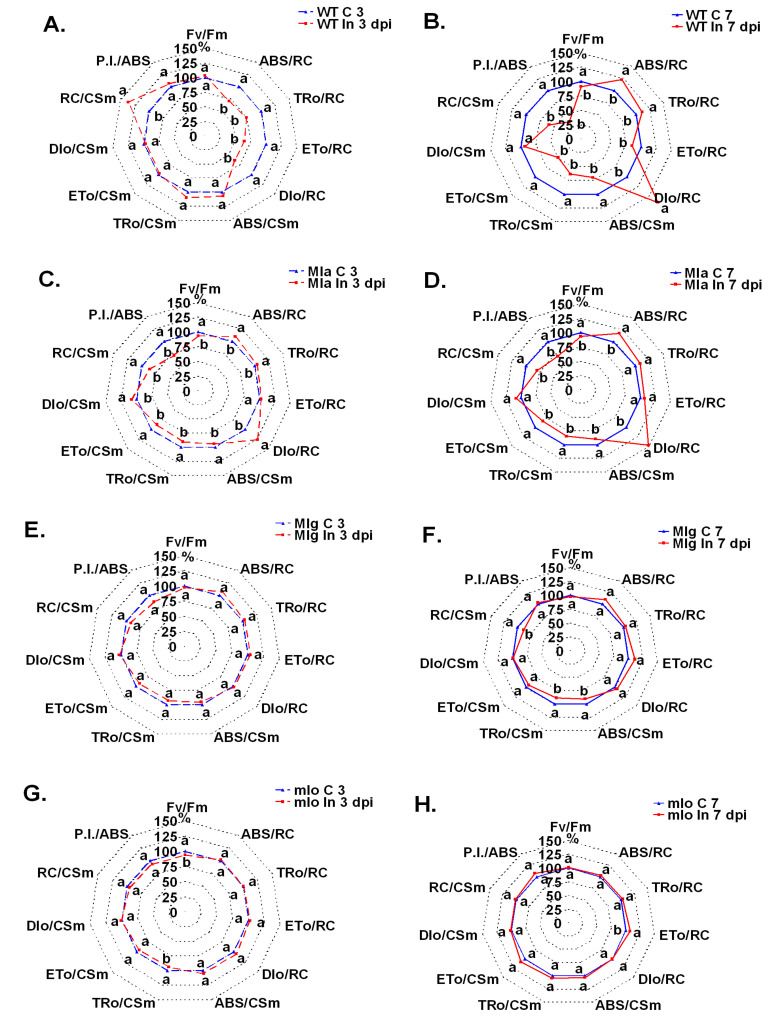
Photosystem II efficiency in powdery mildew-inoculated near-isogenic barley leaves at 3 and 7 days post-inoculation (dpi) as compared to respective healthy control leaves (C 3 or C 7). Changes of 11 photosythetic parameters are presented in spider graphs as percentages of non-inoculated controls (100 %). Panels: (**A**), wild type Ingrid inoculated at 3 dpi vs. control; (**B**), wild type Ingrid inoculated at 7 dpi vs. control; (**C**), Mla inoculated at 3 dpi vs. control; (**D**), Mla inoculated at 7 dpi vs. control; (**E**), Mlg inoculated at 3 dpi vs. control; (**F**), Mlg inoculated at 7 dpi vs. control; (**G**), mlo inoculated at 3 dpi vs. control; (**H**), mlo inoculated at 7 dpi vs. control. Abbreviations: C, control; In, inoculated; WT, wild type Ingrid. Source data are available in Table S1. Values marked with the same letters are not different according to Duncan’s test (*p* ≤ 0.05), *n* = 15.

Powdery mildew inoculation of Ingrid leaves caused disturbances in PSII efficiency and these effects were already observed at 3 dpi, when no disease symptoms were visible. Ingrid is susceptible to powdery mildew so the PSII efficiency progressively declined from 3 dpi to 7dpi due to pathogen development and to decreasing assimilation surface of leaf blades (Table S1; Figure 4). At the early stage of infection (3 dpi) mainly decreases in specific energy fluxes were noted that were calculated per reactive center (RC), such as the energy absorbed by antennas (ABS/RC), the energy transferred to RC (TRo/RC) and the energy flow to electron transport chain (ETo/RC) (Figure 4A). However, these lower (than in control) values of specific energy fluxes were not accompanied by lower phenomenological energy fluxes (per excited cross section CS [per active measured leaf area]) probably due to higher (than in control) density of the active reaction centers per excited cross-section (RC/CS). Measurements conducted at 7dpi on leaves showing visible disease symptoms revealed 22% and 11% increases of ABS/RC and TRo/RC, respectively (Figure 4B), but it did not help to improve the transfer of energy to electron transport chain (ETo/RC).

The ETo/RC value was 16% lower in infected leaves than in non-infected control due to drastically increased dissipation of energy per RC (67%). Moreover, decrease of density of active RCs per excited cross section (RC/CSm) was probably the cause of disturbed energy flow per cross section as shown by diminished values of phenomenological parameters (ABS/CSm, TRo/CSm, ETo/CSm) (Figure 4B). At 7 dpi, one of the most important parameters, ETo/CSm was lowered by 51% in comparison to healthy control. Values of the P.I._ABS_ parameter, which shows that overall performance of PSII, decreased only at 7 dpi but by as much as 65%. Changes in the pattern of energy fluxes of PSII (3 dpi vs. 7 dpi) were completely different between the Mla line (Figure 4C,D) and wild type Ingrid (Figure 4A,B). In Mla line, pathogen spreading was inhibited due to hypersensitive reaction at an early stage of inoculation. The development of necrotic spots (Figure 1) probably had an impact on photosynthesis already at 3 dpi. In Mla leaves, P.I._ABS_, ABS/CSm, TRo/CSm, ETo/CSm decreased already at 3 dpi and practically identical data were obtained at 7 dpi (Figure 4C,D). In Mlg and mlo plants, the changes in PSII performance were only subtle, although sometimes statistically significant (Figure 4E–H).

Kalaji et al. [32] described differences in shape of fluorescence curves and selected fluorescence parameters in wheat plants exposed to particular abiotic stressors. For example, high temperature stress decreased Fm and increased Fo, NaCl stress lowered Fo while drought stress did not change Fo. Our results show that biotic stress caused by pathogen, did not change Fo in all studied genotypes (Figure S1). Values of Fm were lowered relatively strongly in WT and a little less in Mla or Mlg plants. Fm remained unchanged in the highly resistant mlo line (Figure S1). Pathogen infections, especially those causing leaf blade damage, lower the intensity of photosynthesis including the efficiency of light reactions of photosynthesis, which can be measured by fluorescence methods. This is the reason that noninvasive methods of photosynthetic measurements are proposed for presymptomatic detection of infection. Earlier studies of Kuckenberg et al. [33] and Brugger et al. [34] showed that imaging fluorescence of whole leaves was useful for presymptomatic detection of powdery mildew infection in wheat and barley. Our measurements of PSII efficiency, (fast kinetic of chlorophyll *a* fluorescence, the so called JIP test), provides also valuable information. This method has already been used in studies devoted to plant-virus interactions [35,36,37] and plant-bacterium interactions [38,39] and these methods seem to be useful also for fungi-plant interaction.

Our results allowed the characterization of physiological leaf properties and photosynthetic efficiency in powdery mildew inoculated leaves of the barley cultivar Ingrid and its three NILs. Beyond getting new knowledge regarding physiological changes in inoculated lines of three types of resistance, we could confirm that two non-invasive methods, particularly fast kinetics of chlorophyll *a* allowed the presymptomatic detection of powdery mildew infection in barley leaves.

### 2.4. Leaf Hormonal Homeostasis after Powdery Mildew Infection

Plant hormones play important roles not only in regulating developmental processes but also in signalling networks involved in plants’ responses to a wide range of biotic stresses [40,41]. The disturbed hormonal balance can be seen in dwarfing or giant growth of plants, appearance of galls, tumors, leaf deformations or “green islands” on the leaf. In addition to the known biotic stress hormones such as salicylic acid (SA), jasmonates (JA) and ethylene (ET), other hormones such as abscisic acid (ABA), auxins, gibberellins (GA), cytokinins (CK), and brassinosteroids (BR) are also implicated in plant defence signalling pathways [42]. However, their role in plant defence (especially to biotrophic pathogens like powdery mildew or rusts) is less well studied [43,44].

In our experiment we analyzed the foliar level of 23 hormones in powdery mildew-inoculated and control leaves of the barley cultivar Ingrid and its NILs. The investigated hormones were divided into four groups; cytokinins, auxins, gibberellins and “stress hormones”, which involved ABA, (±)-*cis,trans*-abscisic acid glucosyl ester (ABAGlc), JA and SA. In healthy leaves of the near-isogenic barley lines harboring various resistance genes the hormone levels were most often similar to Ingrid leaves, or sometimes slightly reduced. However, after inoculation, in most the cases the amount of individual hormones increased or did not change.

#### 2.4.1. Cytokinins

**Figure 5:**
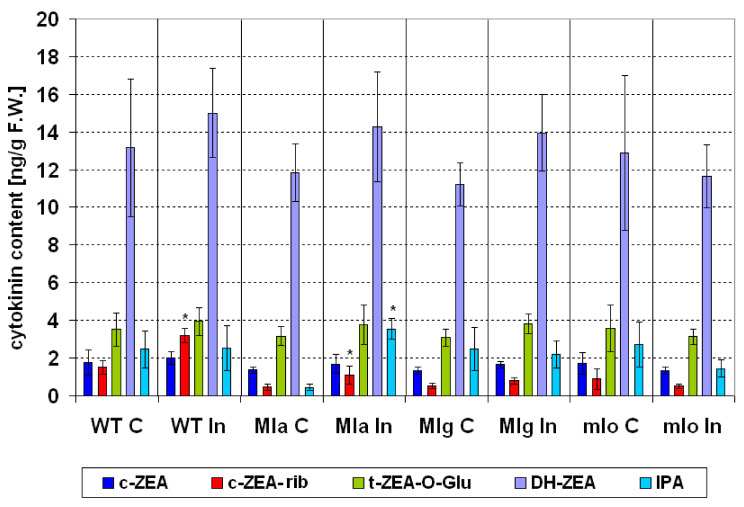
Changes in the contents of five cytokinins in leaves of the susceptible barley cultivar Ingrid and its three near-isogenic lines Mla, Mlg, and mlo at seven days following powdery mildew inoculation (7 dpi). Abbreviations: C, control; In, powdery mildew inoculated; WT, wild type cultivar Ingrid. Investigated hormones: *cis*-zeatin (c-ZEA, active form), *cis*-zeatin riboside (c-ZEA-rib, transport form), *trans*-zeatin-O-glucoside (t-ZEA-O-Glu, degradation form), dihydrozeatin (DH-ZEA, transport form) and N6-isopentenyladenine (IPA, precursor form). Means of five independent repetitions ± SD are shown. The symbol * shows significant differences between inoculated and control leaves (*p* < 5%, Student *t* test) for each genotype (and for particular cytokinin) separately.

In leaves of non-inoculated, control plants the amount of *cis*-zeatin riboside (c-ZEA-rib, cytokinin transport form) was lower in Mla and Mlg than in Ingrid. Similarly, the amount of N6-isopentenyladenine (IPA, precursor form) in Mla was lower as compared to Ingrid, while the levels of all other cytokinins were similar to each other (Figure 5). It is noteworthy that among the cytokinins, which play a significant role in the “green island” phenomenon [45,46], the amount of c-ZEA-rib (cytokinin transport form) significantly increased in inoculated susceptible Ingrid leaves, and in the hypersensitive Mla plants (Figure 5), which is in accordance with previous findings [47]. The amount of IPA significantly increased only in the inoculated leaves of Mla (Figure 5). Here it is also worth recalling that cytokinins play a important role in suppressing virus-and bacterium-induced hypersensitive reactions [48,49]. No significant other changes were found in cytokinin contents of barley lines after powdery mildew inoculation.

#### 2.4.2. Auxins

**Figure 6:**
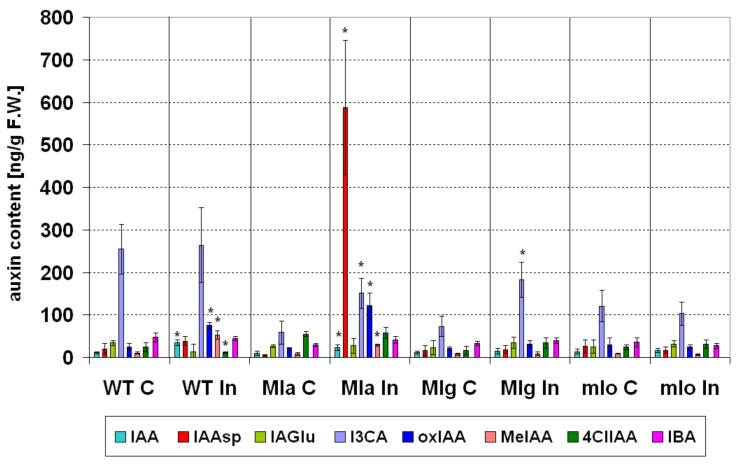
Changes in the contents of eight auxins in leaves of the susceptible barley cultivar Ingrid and its three near-isogenic lines Mla, Mlg, and mlo at seven days following powdery mildew inoculation (7 dpi). Abbreviations: C, control; In, powdery mildew inoculated; WT, wild type cultivar Ingrid. Investigated hormones: indole-3-acetic acid (IAA, active form), indole-3-acetyl-L-aspartic acid (IAAsp, degradation form), indole-3-acetyl-L-glutamic acid (IAGlu, degradation form), indole-3-carboxylic acid (I3CA, degradation form), oxindole-3-acetic acid (oxIAA, degradation form), indole-3-acetic acid methyl ester (MeIAA, precursor form), 4-chloroindole-3-acetic acid (4ClIAA, active form) and indole-3-butyric acid (IBA, precursor form). Means of five independent repetitions ± SD are shown. The symbol * shows significant differences between inoculated and control leaves (*p* < 5%, Student *t* test) for each genotype (and for particular auxin) separately.

Similar to cytokinins, also in case of auxins there were only small differences between healthy Ingrid, Mla, Mlg and mlo lines (Figure 6). Little lower contents of indole-3-carboxylic acid (I3CA, auxin degradation form) and indole-3-butyric acid (IBA, auxin precursor form) were measured in Mla, Mlg and mlo lines. As mentioned the I3CA is a IAA degradation product participating in maintaining of auxin homeostasis. There is even reported a mutual balance between I3CA and IBA [50]. A higher content of 4-chloroindole-3-acetic acid (4ClIAA, active form) was found in Mla as compared to Ingrid (Figure 6). Generally, in comparison to cytokinins, changes in auxin homeostasis after powdery mildew inoculation were quite extended and concerned almost all tested lines and few different auxins (Figure 6). Powdery mildew inoculation significantly elevated the contents of indole-3-acetic acid (IAA, active form), indole-3-acetic acid methyl ester (MeIAA, precursor form) and oxindole-3-acetic acid (oxIAA, degradation form) in Ingrid and Mla lines (Figure 6). Interestingly, another biotrophic pathogen, the stem rust fungus *Puccinia graminis* f. sp. *tritici* also stimulated the accumulation of IAA in wheat [51]. Elevated IAA level is considered as part of defence mechanism and auxins have fungistatic effect [52]. Exogenous auxin administration in low concentration increased the growth of *Fusarium delphinoides* (a chick pea pathogen), but higher auxin concentration inhibited fungal growth [53]. In this context, beyond elevated IAA level, significantly higher level of MeIAA in inoculated Ingrid and Mla line is also intriguing. 

According to Yang et al. [54], MeIAA itself is not an active auxin, but since it is less polar than IAA it can better diffuse across membranes. In this case the transport of IAA (as MeIAA) to shorter or longer distances may be enhanced and MeIAA may be hydrolyzed back to the active form IAA at target places [54]. As for an other auxin, oxIAA that also significantly accumulated in inoculated Ingrid and Mla, a previous report already described higher oxIAA levels in powdery mildew inoculated barley cultivar Delisa and its brassinosteroid-deficient mutant [25]. OxIAA is a physiologically inactive or slightly active product of IAA oxidation and its concentration is elevated as a reaction to increasing IAA levels [55]. In our inoculated Ingrid and Mla plants the elevated oxIAA level was probably also related to elevated IAA levels. To summarize, elevated levels of IAA and MeIAA may be a part of defense mechanisms or pathogenesis in Ingrid and Mla plants.

Elevated level of auxin conjugates (like indole-3-acetyl-L-aspartic acid (IAAsp, degradation form)) in some cases may be also caused by the pathogen [56]. Intriguingly, in our experiments an extremely and significantly increased IAAsp content was measured in the inoculated Mla line (Figure 6). Conjugation of IAA with aspartic acid, to produces indole-3-acetyl aspartic acid (IAAsp), is considered as irreversible and is one of the auxin catabolic pathways [57]. However, according to some studies, IAAsp may be a player in pathogenesis. In roots of *Plasmodiophora brassicae*-infected Chinese cabbage, the level of IAAsp was dramatically enhanced while the free IAA level increased only slightly [58]. A dramatic accumulation of IAAsp was also observed in *Arabidopsis thaliana* infected with *Botrytis cinerea* [56]. According to González-Lamothe et al. [56] this fungal pathogen influences the plant auxin metabolism and elicits the accumulation of a conjugated IAAsp. It was interpreted as a mechanism for promotion of plant susceptibility by pathogen through the conjugation of an active auxin. IAAsp is beneficial to the pathogen also because it increases disease spreading by regulating the transcription of virulence genes [56]. In the light of this report we can suggest a theory that in inoculated Mla where the active IAA level was about 5-fold higher than in control, an increase of IAAsp concentration might be pathogen-stimulated in purpose to weaken antifungal defense in this resistant genotype. Interestingly, in the susceptible wild type Ingrid plants similar increases of IAA and IAAsp levels were noticed, but to a much extent than in Mla (Figure 6).

Powdery mildew inoculation of Mla and Mlg lines that showed hypersensitive reaction led to significantly elevated level of I3CA (auxin degradation form) (Figure 6). We observed earlier the same reaction in powdery mildew-inoculated barley cultivar Delisa and its brassinosteroid-deficient mutant [25]. Interestingly, both Delisa and its mutant, similar to the Mlg and mlo lines used in the present study (Figure 1), showed no visible disease symptoms [25]. I3CA interplaying with β-aminobutyric acid may be a mediator of priming against necrotrophic fungal pathogen *Plectosphaerella cucumerina* [59]. The role of I3CA in shaping antifungal plant resistance in barley requires deeper studies, especially that I3CA level was generally lower in resistant lines Mla, Mlg and mlo than in Ingrid (both in control and inoculated plants (Figure 6).

Additionally, inoculated Ingrid leaves were also characterized by significantly decreased levels of 4ClIAA (auxin active form) (Figure 6). Earlier we found increased level of chlorinated auxins in powdery mildew inoculated barley leaves [25], but Delisa and its brassinosteroid-deficient mutant showed no visible disease symptoms, contrary to Ingrid. Knowledge about the physiological role of chlorinated auxis is very poor [60], and their function in pathogenesis remains to be explained.

In summary, the most extensive changes in contents of various auxins were found in those inoculated plants, which showed massive or slight visible symptoms (Ingrid and Mla, respectively). Only small changes in auxin contents were noticed in Mlg (mainly in I3CA). The auxin profiles of inoculated and control mlo plants did not differ significantly.

#### 2.4.3. Gibberellins

**Figure 7:**
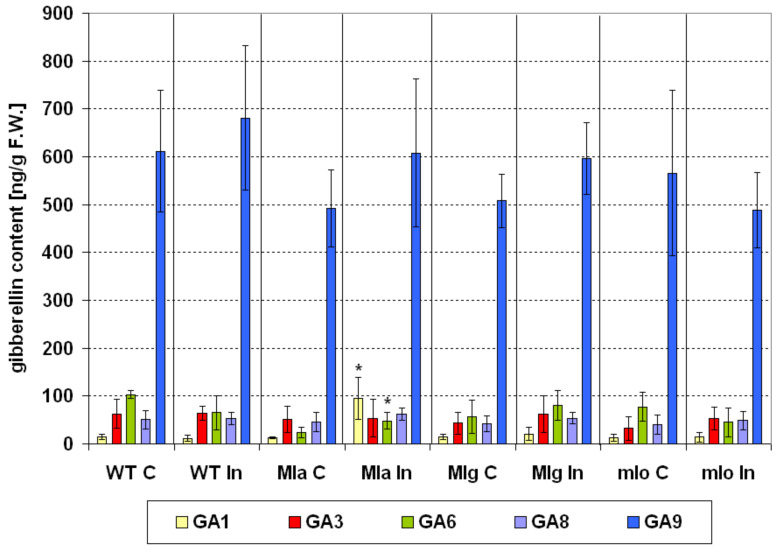
Changes in the contents of five gibberellins in leaves of the susceptible barley cultivar Ingrid and its three near-isogenic lines Mla, Mlg, and mlo at seven days following powdery mildew inoculation (7 dpi). Abbreviations: C, control; In, powdery mildew inoculated; WT, wild type cultivar Ingrid. Investigated hormones: gibberellin A_1_ (GA1, active form), gibberellic acid (GA3, active form), gibberellin A_6_ (GA6, active form), gibberellin A_8_ (GA8, degradation form), and gibberellin A_9_ (GA9, precursor form). Means of five independent repetitions ± SD are shown. The symbol * shows significant differences between inoculated and control leaves (*p* < 5%, Student *t* test) for each genotype (and for particular gibberellin) separately.

In healthy barley, gibberellins were present in similar amount in all tested genotypes with the one exception of gibberellin A_6_ (GA6, active form)–slightly lower in Mla lines as compared to Ingrid (Figure 7). Upon inoculation, a significant increase of gibberellin A_1_ (GA1, active form) level and a slight but significant elevation of GA6 in Mla leaves were only observed (Figure 7). Similar reactions were noted in our previous studies especially regarding GA1 [25]. The barley cultivar Delisa and its brassinosteroid mutant showed an increased level of GA1 after powdery mildew inoculation, although the GA6 content did not change in the mutant while it increased very slightly in Delisa. Generally, the role of gibberellins in plant-pathogen interactions is significantly lesser explored in comparison to other hormone groups [61]. Gibberellins are supposed to play a role in rice immunity to *Pythium graminicola.* The gibberellic acid (GA3, active form) enhanced the resistance of rice to *P. graminicola,* while lowering GA levels by using GA inhibitor caused an increased disease susceptibility [62]. Among our barley lines, the profile of gibberellins was generally very similar with only a small exception (Mla) and this profile did not change even in the susceptible Ingrid after powdery mildew infection. Presumably gibberellins do not play a pivotal role in the powdery mildew resistance of barley.

#### 2.4.4. Stress Hormones

**Figure 8:**
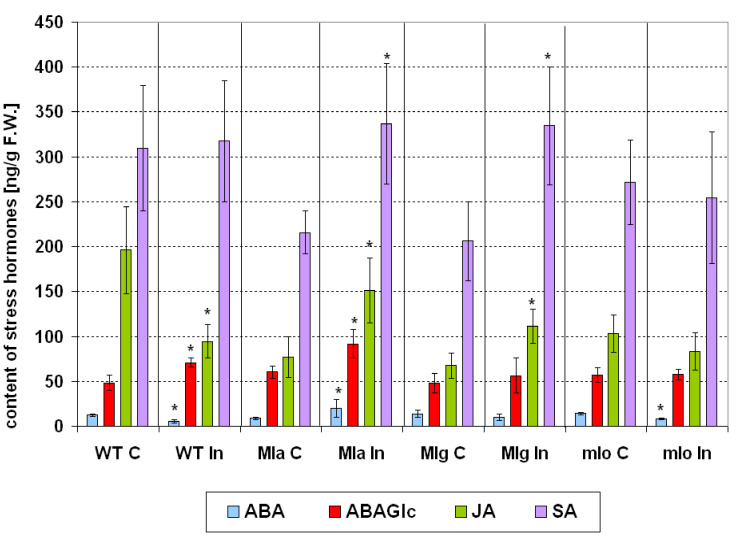
Changes in the contents of four stress hormones in leaves of the susceptible barley cultivar Ingrid and its three near-isogenic lines Mla, Mlg, and mlo at seven days following powdery mildew inoculation (7 dpi). Abbreviations: C, control; In, powdery mildew inoculated; WT, wild type cultivar Ingrid. Investigated hormones: (±)-*cis, trans*-abscisic acid (ABA, active form), (±)-*cis, trans*-abscisic acid glucosyl ester (ABAGlc, inactive form), (±)-jasmonic acid (JA, active form), and salicylic acid (SA, active form). Means of five independent repetitions ± SD are shown. The symbol * shows significant differences between inoculated and control leaves (*p* < 5%, Student *t* test) for each genotype (and for particular hormone) separately.

When comparing healthy barley lines, lower amounts JA and SA were observed in Mla and Mlg lines as compared to Ingrid (Figure 8). JA level was also lowered in mlo line in comparison to Ingrid. Powdery mildew inoculation caused strong changes in stress hormone levels of barley lines. Inoculated leaves of Mla plants showed significantly increased levels of JA, SA, ABA (active forms) and ABA conjugate (ABAGlc; inactive form), while inoculated Mlg leaves had significantly elevated JA and SA contents in comparison to healthy (Figure 8). Elevated levels of JA and SA in resistant Mla and Mlg lines can be justified/expected because both hormones are usually a part of resistance mechanisms, their contents increase in infected tissues and their exogenous application reduces symptoms of fungal diseases [63,64,65,66]. However, our results are not in agreement with findings of Hückelhoven et al. [6], who did not detect any elevated SA level in barley cultivar Pallas and in its *mlo5*-, *Mlg*-, and *Mla12*-backcross lines after powdery mildew infection. The reason could be partly connected to different genetic background of wild type cultivars (cv Pallas vs. cv. Ingrid).

Interestingly however, in susceptible Ingrid leaves the JA content significantly decreased after inoculation in comparison to non-inoculated control (Figure 8). This fact can be discussed in lights of findings of Patkar et al. [67]. The rice blast fungus *Magnaporthe oryzae* can influence JA level in host plants by producing JA derivatives and secreting an enzyme that converts endogenous JA into hydroxylated JA. This hydroxylated JA form prevents the induction of JA signaling and disturbs JA-dependent defense in plant [67]. In wild type Ingrid, such phenomenon could be one of the reasons of susceptibility to powdery mildew and the appearance of massive disease symptoms. Still, another possibility is that reduction of endogenous JA levels in the susceptible genotype is a result of JA conjugation and JA-Ile (jasmonyl-isoleucine) production. That would comply with the results of Schuman et al. [68] obtained for tobacco, where the susceptible genotype had lower JA content. This hypothesis should be verified in further experiments. It is however a little surprising that originally in non-inoculated susceptible Ingrid leaves the level of JA was much higher than in the powdery mildew resistant lines (Figure 8). Furthermore, interestingly the susceptible wild type Ingrid showed a significantly higher amount of ABAGlc but significantly lower ABA content after inoculation (Figure 8). The conjugated form ABAGlc can however serve as an ABA reservoir and ABA is released by deconjugation of this hydrolyzable conjugate in reaction to stress [69]. The eleveted amount of ABAGlc together with lower ABA content in infected Ingrid may inform about a disturbance in deconjugation and potentially lowers of the resistance to pathogen. Changes in ABA and ABAGlc patterns upon inoculation were different in Mla from Ingrid, and rather similar to our earlier observations with powdery mildew-inoculated, resistant barley cultivar Delisa [25]. In leaves of both Mla and Delisa, the inoculation increased the concentration of ABA and its hydrolysable conjugate ABAGlc. According to the literature, ABA is a positive regulator of barley basal resistance to powdery mildew [70]. ABA stimulates the resistance to attempted *Bgh* penetration in wild type barley and the transcription factor *HvNAC6* acts as a regulator of ABA-mediated defense responses to maintain an effective basal resistance against this pathogen [70]. Thus we can propose that in Mla ABA type of resistance seems to be one of important players in defense while lowered ABA content in wild type Ingrid may be one of reasons its susceptibility. Finally, the lack of differences in JA and SA levels between control and inoculated mlo plants (Figure 8) can be interpreted as meaning that some unknown mechanisms blocked the pathogen at a stage early enough not to activate responses connected with these hormones. This presumption regarding inoculated mlo plants is true also for other aforementioned group of hormones. The only exception however was slightly (but significantly) lowered content of ABA in inoculated mlo plants (than in not inoculated), but this phenomenon rather was connected with the strongest disturbances in water relations (see WBI values of inoculated plants, Figure 3) than with direct pathogen effect. Generally, as for measured in this work other physiological changes (like PSII efficiency) and their connection to hormonal homeostasis, we cannot find a clear picture. Hormones (especially those responsible for growth like cytokinins or auxins) can stimulate widely understood photosynthetic processes especially under stress [71]. In our work however PSII efficiency after inoculation was little lower in Mla plants than in other resistant genotypes (Table S1, parameter P.I._ABS_) although Mla plants, in reaction to pathogen, had rather increased level of some auxins and cytokinins while inoculated Mlg or mlo plants did not.

In summary, our data on hormonal changes in powdery mildew inoculated barley lines confirmed that not only SA, JA or ethylene, but other hormones and their derivatives can play important roles in disease resistance or susceptibility to a biotrophic pathogen. Development of the analytical techniques makes the simultaneous detection of many plant hormones and hormone derivatives possible. Detailed analysis of changing hormone levels after pathogen inoculations is a first step to a deeper knowledge about the biological significance of all hormone classes and their complex interplay in disease resistance.

### 2.5. Concluding Remarks

We characterized leaf physiological properties as well as photosynthetic and hormonal changes in susceptible wild type barley cultivar Ingrid and its near-isogenic lines carrying various resistant genes (*Mla*, *Mlg* and *mlo*). The aim of our studies was to describe some of physiological mechanisms accompanying various types of barley resistance to powdery mildew. Generally *Bgh* inoculated resistant lines especially mlo and Mlg were characterized by high efficiency of light reactions of photosynthesis expressed both by parameters of fluorescence (such as P.I._ABS_ or Fv/Fm) as well as leaf reflectance parameter PRI (which can be partly related to PSII quantum yield). These changes can be connected to the lack of visible symptoms i.e., undamaged leaf blades (Figure 1). On the other hand, it is also possible that mobilization of photosynthesis (higher energy conservation) may be part of protective mechanisms against *Bgh*, especially that values of PRI for non-inoculated controls were much lower at the same time. Leaf blade injuries in susceptible Ingrid are probably connected to decreased PSII activity. A lesser but still significant decrease of PSII activity was measured in Mla with visible hypersensitive necrotic spots. In addition, chlorophyll *a* fluorescence measurements allowed the presymptomatic detection of infection (at 3 dpi) in these genotypes. Hormone profiles of inoculated and control mlo plants generally did not differ significantly, probably due to the undameged inoculated leaves. Contrary to mlo, the two resistant lines Mlg and Mla were characterized by altered hormonal homeostasis regarding both growth hormones and stress hormones. The hormonal changes were however much more pronounced in Mla, where not only the levels of SA, JA, ABA but also those of several auxins, gibberellins and cytokinins markedly changed. Inoculation of susceptible Ingrid wild type plants influenced also the hormonal homeostasis and directions of these changes were slightly different from those observed in Mla plants.

## 3. Material and Methods

### 3.1. Plant Material and Experimental Design

The susceptible barley (*Hordeum vulgare* L.) cultivar Ingrid (wild type) and its three near-isogenic lines carrying the *Mla12, Mlg* or *mlo5* resistance genes to powdery mildew were selected for the experiments. Abbreviations used in text for genotypes carrying these mutations are respectively Mla, Mlg and mlo. Seeds were sown on wet paper in Petri dishes and kept three days in darkness (24 °C). Germinated seeds were moved to pots with soil (pot size: 13 cm × 13 cm × 13 cm; number of plants per pot: 7–10). Plants continued to grow in glasshouse at natural conditions of light and a photoperiod characteristic for May/June (Central-European region). Ten-days-old plants were divided into two groups. Plants of one group were not inoculated controls. Plants of the second group were inoculated with powdery mildew (*Blumeria graminis* f. sp. *hordei* A6 race, *Bgh*). There is a typical gene for gene interaction; Bgh race 6 interacts with *Mla*, *Mlg* and *mlo* genes as described in details in the literature, where authors used this race of barley powdery mildew [5,6,10,72]. After three and seven days post inoculation (dpi) non-invasive leaf reflectance and PSII efficiency measurements were made. Samples for hormonal analysis were collected at seven dpi. Measurements and sample collection were made simultaneously for leaves of not inoculated (control) plants at the corresponding time points. Photos were made seven dpi.

### 3.2. Measurements of Leaf Reflectance and Reflection Parameters

Reflectance was measured on powdery mildew-inoculated barley leaves at 3 and 7 dpi and simultaneously on leaves of not inoculated, control plants at the corresponding time points. Measurements of reflection were always made on top side of the leaf by a CI-710 Miniature Leaf Spectrometer (wavelength range: 400–950 nm; sensitivity: 130 photons/count at 400 nm, 60 photons/count at 600 nm; optical resolution: ~0.3–10.0 nm FWHM [Full width at half maximum-grating dependent]; producer: CID Bio-Science, Camas, WA, USA). Other technical details of the measurements are also described in the paper of Janeczko et al. [73]. Since the reflectance values of not inoculated control leaves were similar at time points corresponding to 3 and 7 dpi they were summed up and shown as one control on Figure 2. Based on the reflection curves obtained at 7 dpi, selected reflection parameters were calculated: CRI = (R_510_^−1^ − R_550_^−1^) × R_800_ [74], this parameter informs about carotenoids content; SIPI = (R_800_ − R_445_)/(R_800_ + R_680_), this parameter is associated to the relation of carotenoids to chlorophyll *a* [11]; WBI = R_900_/R_970_ [10], water band index parameter informs about leaf water status; PRI = (R_531_ − R_570_)/(R_531_ + R_570_), this parameter (photochemical reflectance index) has been found to be well correlated to non-photochemical quenching (NPQ) and photosynthetic light use efficiency (LUE) at both leaf and canopy levels [19]. In the equations, R_x_ means reflectance intensity at a specific x wavelength. Measurements were done in 10 repetitions per treatment (1 repetition = 1 leaf from one plant).

### 3.3. Fast Kinetics of Chlorophyll A Fluorescence

The fast fluorescence kinetics of chlorophyll *a* was used for the estimation of PSII efficiency. Measurements of dark-adapted first leaves were done using a Plant Efficiency Analyser (Hansatech Ltd., King’s Lynn, UK). Technical details were previously described by Skoczowski et al. [38]. Briefly: leaf dark adaptation: 30 min. with to use of clips with a 4 mm diameter hole; changes in fast fluorescence were registered during illumination in time 10 μs to 1 s, after this time the frequency of measurements was reduced; the excitation light intensity of 3 mmol m^−2^ s^−1^ (peak 650 nm). Measurements were done between 10.00–12.00 a.m., on plants cultured in natural light conditions in greenhouse (May/June-Central-European region). Fluorescence curves recorded for leaves were used for reading/calculating of the following technical parameters described in the paper of Strasser et al. [75]: Fo—fluorescence intensity at 50 µs (all PS II reaction centres (RC) are open), Fm—maximal fluorescence intensity (all RC are closed), F_300_—fluorescence intensity at 300 µs, F_J_—fluorescence intensity at 2 ms, V_J_ (relative variable fluorescence) = (F_J_ − Fo)/(Fm − Fo), Mo = 4 × (F_300_ − Fo)/(Fm − Fo), Fv—the difference between maximum fluorescence and minimum fluorescence. Next, calculations of ratio Fv/Fm (the maximum quantum yield of photosystem II primary photochemistry) as well as specific and phenomenological fluxes were done. Specific fluxes per reactive center (RC): energy absorption by the antenna system [ABS/RC = M_0_ × (1/V_J_) × (1/(Fv/Fm)], energy trapped in a reaction center (TRo/RC = M_0_ × (1/V_J_)), energy flux to electron transport chain (ETo/RC = M_0_ × (1/V_J_) × (1 − V_J_)), energy dissipated as heat (DIo/RC = (ABS/RC) − (TRo/RC)); phenomenological fluxes (per CS-cross section [i.e., per active measured leaf area]): energy absorption by the antenna system (ABS/CSm = Fm), energy trapped in a reaction center (TRo/CSm = Fv/Fm × (ABS/CS)), energy flux to electron transport chain (ETo/CSm = (Fv/Fm) × (1 − V_J_) × Fm), energy dissipated as heat (DIo/CSm = (ABS/CS) × (TRo/CS)); reactive centers concentration (or density) per excited cross section of samples RC/CS = (ABS/CSm)/(ABS/RC); performance index which shows generally PSII efficiency (P.I._ABS_). More detailed explanations and equations of these parameters can be found in the paper of Strasser et al. [75]. Measurements were done in 15 repetitions per treatment (1 repetition = 1 leaf from one plant).

### 3.4. Hormonal Assays

Hormone assays were based on a modified method of Dziurka et al. [76], and the protocol of Dobrev and Kaminek [77]. Samples of first leaves (0.5 g fresh weight) were collected from 3–5 plants and homogenized with ceramic beads (Bead Ruptor Elite, Omni International, Kennesaw, GA, USA). Then internal standard (ISTD) mixture was added and samples were extracted in a mixture of methanol:water:formic acid (15:4:1 v/v). Plant extracts were evaporated under N_2_, resuspended in 3% MeOH in 1M HCOOH, and purified on SPE-Bond Elut Plexa PCX columns (30 mg, 1 mL, Agilent, Santa Clara, CA, USA) according to manufacturer procedure. The analysis of hormones was performed on an Ultra High-Performance Liquid Chromatography apparatus (Agilent Infinity 1260, Agilent, Waldbronn, Germany) coupled to a triple quadruple mass spectrometer (6410 Triple Quad LC/MS, Agilent, Santa Clara, CA, USA) with electrospray ionization (ESI). Sample separation was achieved by an Ascentis Express RP-Amide analytical column (2.7 μm, 2.1 mm × 150 mm; Supelco, Bellefonte, PA, USA) in gradient mode of H_2_O vs. ACN, modified with 0.01% HCOOH, at 0.5 ml/min. The following hormones were analyzed: cytokinins—*cis*-zeatin (c-ZEA; active form), *cis*-zeatin riboside (c-ZEA-rib; transport form), *trans*-zeatin-O-glucoside (t-ZEA-O-Glu; degradation form), dihydrozeatin (DH-ZEA; transport form), N6-isopentenyladenine (IPA; precursor form), [^15^N_4_]dihydrozeatin (DH-ZEA-N15) used as ISTD; auxins—indole-3-acetic acid (IAA; active form), indole-3-acetyl-L-aspartic acid (IAAsp; degradation form), indole-3-acetyl-L-glutamic acid (IAGlu; degradation form), indole-3-carboxylic acid (I3CA; degradation form), oxindole-3-acetic acid (oxIAA; degradation form), indole-3-acetic acid methyl ester (MeIAA; precursor form), 4-chloroindole-3-acetic acid (4ClIAA; active form), indole-3-butyric acid (IBA; precursor form) and [^2^H_5_]indole-3-acetic acid (IAA-D5) and [^2^H_5_]indole-3-acetic acid methyl ester (MeIAA-D5) used as ISTD; gibberellins ‒ gibberellin A_1_ (GA1; active form), gibberellic acid (GA3; active form), gibberellin A_6_ (GA6; active form), gibberellin A_8_ (GA8; degradation form), gibberellin A_9_ (GA9; precursor form), and [^2^H_2_]gibberellin A_1_ (GA1-D2), [^2^H_2_]gibberellin A_6_ (GA6-D2); [^2^H_2_]gibberellin A_8_ (GA8-D2) used as ISTD; stress hormones ‒ (±)-*cis, trans*-abscisic acid (ABA; active form), (±)-*cis, trans*-abscisic acid glucosyl ester (ABAGlc; inactive form/degradation form), (±)-jasmonic acid (JA; active form), salicylic acid (SA; active form) and [^2^H_6_]*cis*, *trans*-abscisic acid (ABA-D6), [^2^H_4_]salicylic acid (SA-D4), [^2^H_5_]jasmonic acid (JA-D5) used as ISTD. Authentical hormones and heavy-labeled standards were purchased at Olchemim (Olomouc, Czech Republic) except JA-D5 supplied by CND Isotopes (Pointe-Claire, QC, Canada). Identification and quantitation were based on multiple reaction monitoring (MRM) transitions, using linear calibration curves obtained for pure standards and taking account recoveries of ISTD (details in Supplementary Materials—Table S2). The measurements were done in five repetitions for each treatment (one repetition = leaves taken from 3–5 plants from different pots).

### 3.5. Statistical Calculations

Statistical analyses (ANOVA, post hoc test) were made by using Statistica 13.1 (StatSoft, Inc., Tulsa, OK 74104, USA). The number of repetitions for particular measurements was given in particular method descriptions. The Duncan’s test was used to compare averages (cultivar Ingrid and NILs: Mla, Mlg, mlo) in case of measurements of leaf reflectance and PSII efficiency (chlorophyll *a* fluorescence). Averages of leaf reflectance parameters are presented on Figure 3 ± SD. Average values ± SD of parameters of PSII efficiency are given in Table S1. Average values ± SD of technical fluorescence parameters Fo and Fm are given on Figure S1. Percentage changes of PSII parameters in inoculated plants are shown in Figure 4 in comparison to values obtained for not inoculated control (100%). Statistically significance has been proven sometimes even in case of very small differences between averages on Figure 4, due to high repeatability of results (and low standard deviations); in such cases see also data in Table S1. Results of hormonal analysis are presented as average values ± SD on Figure 5, Figure 6, Figure 7 and Figure 8 where stars show significant differences between inoculated and control leaves (*p* < 5%, Student *t* test) for each genotype (and for particular hormone) separately.

## Supplementary Materials

Supplementary materials can be found at https://www.mdpi.com/1422-0067/21/12/4536/s1. Table S1: Supplementary data. Photosystem II efficiency measured for barley leaves inoculated by powdery mildew (In; 3 and 7 dpi) and also measured at the corresponding time points in plants of not inoculated control (C; respectively 3 and 7 day). WT–wild type Ingrid; Mla, Mlg, mlo–NILs with various resistance genes. Average values ± SD; Table S2: Multiple reactions monitoring (MRM) transitions used for identification and quantitation of the analyzed phytohormones. Data arranged in the elution order. Ion source parameters: positive ion mode (+ESI), capillary voltage 4 kV, gas temperature 350 °C, gas flow 12 l/min, and nebulizer pressure 35 psi; Figure S1: Fluorescence parameters Fo and Fm measured in barley leaves inoculated by powdery mildew (In; 7 dpi) and also measured at the corresponding time points in plants of not inoculated control (C; 7 day). WT–wild type Ingrid; Mla, Mlg, mlo–NILs with various resistance genes. Values marked with the same letters (in pairs WT C 7 and WT In 7 dpi; Mla C 7 and Mla In 7 dpi, Mlg C 7 and Mlg In 7 dpi, mlo C 7 and mlo In 7 dpi) are not different according to Duncan’s test (*p* ≤ 0.05), *n* = 15.
