# Supplementary figures and images for: Powdery Mildew-Induced Hormonal and Photosynthetic Changes in Barley Near Isogenic Lines Carrying Various Resistant Genes
Source: Int J Mol Sci. 2020 Jun 25;21(12):4536. doi: 10.3390/ijms21124536 (PMC7352864; doi:10.3390/ijms21124536)

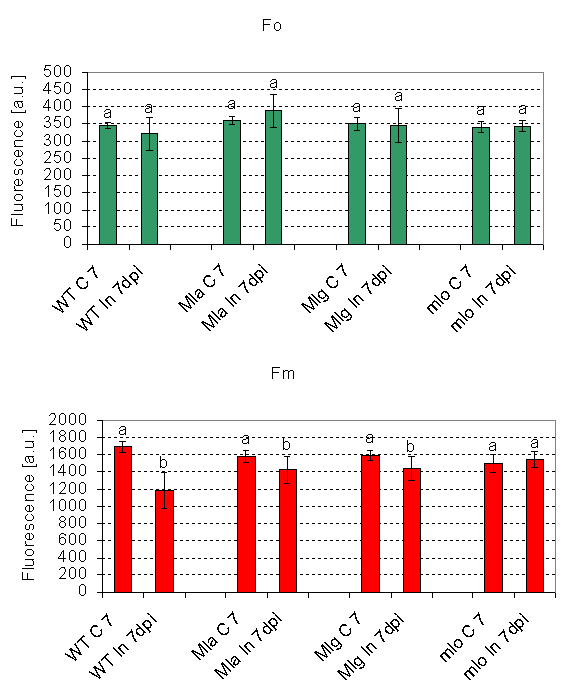

Supplement: Supplementary file 1 [file ijms-21-04536-s001.zip › Figure S1 supplement.tif]
