# Supplementary material for: Powdery Mildew-Induced Hormonal and Photosynthetic Changes in Barley Near Isogenic Lines Carrying Various Resistant Genes
Source: Int J Mol Sci. 2020 Jun 25;21(12):4536. doi: 10.3390/ijms21124536 (PMC7352864; doi:10.3390/ijms21124536)
Supplement: Supplementary file 1 [file ijms-21-04536-s001.zip › Table S1 supplement.doc]

**Table S1.** Supplementary data. Photosystem II efficiency measured for barley leaves inoculated by powdery mildew (In; 3 and 7 dpi) and also measured at the corresponding time points in plants of not inoculated control (C; respectively 3 and 7 day). WT – wild type Ingrid; Mla, Mlg, mlo – NILs with various resistance genes. Average values ±SD.

| Treatment | Fv/Fm | ABS/RC | TRo/RC | ETo/RC | DIo/RC | ABS/CSm | TRo/CSm | ETo/CSm | DIo/CSm | RC/CSm | P.I._ABS_ |
| --- | --- | --- | --- | --- | --- | --- | --- | --- | --- | --- | --- |
| WT C 3 | 0.727±0.038 | 3.70±1.11 | 2.66±0.71 | 1.85±0.67 | 1.03±0.41 | 1387±134 | 1013±149 | 688±85 | 374±21 | 412±148 | 1.72±0.53 |
| WT In 3 dpi | 0.751±0.031 | 2.63±0.18 | 1.97±0.09 | 1.19±0.06 | 0.66±0.12 | 1473±146 | 1110±154 | 672±108 | 363±16 | 565±89 | 1.83±0.51 |
| WT C 7 | 0.796±0.008 | 2.48±0.10 | 1.98±0.07 | 1.16±0.02 | 0.51±0.03 | 1691±65 | 1347±63 | 789±67 | 344±8 | 682±49 | 2.26±0.43 |
| WT In 7 dpi | 0.724±0.033 | 3.04±0.45 | 2.19±0.25 | 0.98±0.12 | 0.85±0.22 | 1177±208 | 856±177 | 390±120 | 321±49 | 394±85 | 0.80±0.35 |
| Mla C 3 | 0.794±0.013 | 2.39±0.15 | 1.90±0.11 | 1.22±0.04 | 0.49±0.05 | 1628±77 | 1294±81 | 832±70 | 334±8 | 684±57 | 2.96±0.54 |
| Mla In 3 dpi | 0.771±0.010 | 2.61±0.15 | 1.99±0.08 | 1.24±0.05 | 0.62±0.09 | 1522±83 | 1162±93 | 728±66 | 360±15 | 587±58 | 2.11±0.38 |
| Mla C 7 | 0.773±0.011 | 2.65±0.15 | 2.04±0.11 | 1.11±0.10 | 0.60±0.05 | 1580±69 | 1221±67 | 665±85 | 359±12 | 599±45 | 1.58±0.38 |
| Mla In 7 dpi | 0.724±0.051 | 3.11±0.68 | 2.22±0.30 | 1.18±0.20 | 0.89±0.39 | 1419±158 | 1032±170 | 554±117 | 387±48 | 478±114 | 1.10±0.45 |
| Mlg C 3 | 0.770±0.021 | 2.73±0.42 | 2.10±0.27 | 1.36±0.26 | 0.63±0.15 | 1541±82 | 1188±94 | 764±58 | 353±16 | 576±91 | 2.28±0.36 |
| Mlg In 3 dpi | 0.757±0.029 | 2.95±1.01 | 2.17±0.55 | 1.41±0.47 | 0.65±0.10 | 1465±159 | 1101±176 | 706±109 | 364±28 | 531±124 | 1.99±0.58 |
| Mlg C 7 | 0.780±0.013 | 2.58±0.13 | 2.01±0.09 | 1.11±0.08 | 0.57±0.06 | 1592±58 | 1242±58 | 685±71 | 349±19 | 620±43 | 1.74±0.41 |
| Mlg In 7 dpi | 0.771±0.019 | 2.81±0.85 | 2.08±0.36 | 1.22±0.27 | 0.73±0.49 | 1443±144 | 1096±170 | 644±119 | 346±49 | 544±125 | 1.80±0.70 |
| mlo C 3 | 0.794±0.012 | 2.43±0.10 | 1.93±0.06 | 1.20±0.03 | 0.50±0.05 | 1614±75 | 1283±78 | 801±68 | 331±8 | 664±39 | 2.67±0.50 |
| mlo In 3 dpi | 0.781±0.016 | 2.49±0.08 | 1.94±0.07 | 1.23±0.04 | 0.53±0.02 | 1572±55 | 1200±94 | 761±64 | 335±9 | 636±35 | 2.51±0.35 |
| mlo C 7 | 0.772±0.014 | 2.55±0.08 | 1.97±0.10 | 1.07±0.05 | 0.58±0.06 | 1494±106 | 1155±100 | 632±89 | 339±15 | 588±64 | 1.72±0.32 |
| mlo In 7 dpi | 0.777±0.015 | 2.61±0.19 | 2.02±0.13 | 1.15±0.07 | 0.58±0.07 | 1542±94 | 1200±91 | 686±81 | 343±16 | 596±66 | 1.84±0.44 |
