# Supplementary material for: Powdery Mildew-Induced Hormonal and Photosynthetic Changes in Barley Near Isogenic Lines Carrying Various Resistant Genes
Source: Int J Mol Sci. 2020 Jun 25;21(12):4536. doi: 10.3390/ijms21124536 (PMC7352864; doi:10.3390/ijms21124536)
Supplement: Supplementary file 1 [file ijms-21-04536-s001.zip › Table S2 supplement.doc]

Table S2. Multiple reactions monitoring (MRM) transitions used for identification and quantitation of the analyzed phytohormones. Data arranged in the elution order. Ion source parameters: positive ion mode (+ESI), capillary voltage 4 kV, gas temperature 350 °C, gas flow 12 l/min, and nebulizer pressure 35 psi.

| Compound | Type of ion | Quantifier transition  (precursor/product ions) | Fragmentor voltage (V) | Collision energy (V) | MRM Start Time (min) |
| --- | --- | --- | --- | --- | --- |
| t-ZEA-O-Glu | [M+H]^+^ | 382.1/202.1 | 142 | 17 | 1.6 |
| c-ZEA | [M+H]^+^ | 220.2/136.3 | 85 | 9 |  |
| DH-ZEA-N15 | [M+H]^+^ | 226.2/140 | 124 | 18 |  |
| DH-ZEA | [M+H]^+^ | 222.2/136 | 124 | 18 |  |
| oxIAA | [M+H]^+^ | 192.2/146.1 | 54 | 9 | 4.0 |
| GA8-D2 | [M-H2O+H]^+^ | 321.2/257.2 | 102 | 9 |  |
| GA8 | [M-H2O+H]^+^ | 319.3/255.2 | 102 | 9 |  |
| c-ZEA-rib | [M+H]^+^ | 352.2/220.3 | 120 | 9 |  |
| IAAsp | [M+H]^+^ | 291.2/130.1 | 54 | 25 |  |
| IPA | [M+H]^+^ | 204.1/148.3 | 90 | 9 | 7.4 |
| IAGlu | [M+H]^+^ | 305.2/130.1 | 58 | 29 |  |
| SA-D4 | [M+H]^+^ | 143.2/125.2 | 80 | 14 | 8.5 |
| SA | [M+H]^+^ | 139.2/121.2 | 80 | 14 |  |
| I3CA | [M+H]^+^ | 161.2/118.1 | 50 | 10 |  |
| IAA-D5 | [M+H]^+^ | 181.1/135.1 | 38 | 14 |  |
| IAA | [M+H]^+^ | 176.1/130.3 | 51 | 9 |  |
| GA3 | [M-H2O+H]^+^ | 329.3/311.3 | 100 | 14 |  |
| GA1-D2 | [M-H2O+H]^+^ | 333.3/287.2 | 58 | 9 | 10.3 |
| GA1 | [M-H2O+H]^+^ | 331.3/285.3 | 100 | 14 |  |
| ABAGlc | [M-H2O+H]^+^ | 409.2/247.1 | 104 | 14 |  |
| GA6-D2 | [M-H2O+H]^+^ | 331.3/258.3 | 104 | 14 | 12.4 |
| GA6 | [M-H2O+H]^+^ | 329.3/283.3 | 104 | 14 |  |
| ABA-D6 | [M-H2O+H]^+^ | 253.4/191.3 | 80 | 14 | 16.7 |
| ABA | [M-H2O+H]^+^ | 247.4/187.2 | 80 | 14 |  |
| 4ClIAA | [M+H]^+^ | 210.4/164 | 80 | 14 | 17.8 |
| MeIAA-D5 | [M+H]+ | 195/134 | 54 | 9 |  |
| MeIAA | [M+H]+ | 190/130 | 54 | 9 |  |
| JA-D5 | [M+H]^+^ | 216.3/153.2 | 80 | 5 | 18.8 |
| JA | [M+H]^+^ | 211.3/151.2 | 80 | 14 |  |
| IBA | [M+H]^+^ | 204.1/186.4 | 69 | 9 |  |
| GA9 | [M-H2O+H]^+^ | 271.3/225.2 | 136 | 13 | 23.1 |
